# TranslAGE: A Unified Platform to Build and Translate Aging Biomarkers into Clinical Surrogate Endpoints

**DOI:** 10.1093/geroni/igaf122.4406

**Published:** 2025-12-31

**Authors:** Raghav Sehgal

**Affiliations:** Yale University, New Haven, Connecticut, United States

## Abstract

Evaluating the effectiveness of lifespan- and healthspan-extending interventions in human clinical trials requires robust surrogate endpoints. Omics-based aging biomarkers hold promise but face limitations: narrow validation efforts, lack of standardization, and population biases. To address these, we developed TranslAGE, a knowledgebase and clinical trial platform that benchmarks aging biomarkers using three key metrics—Responsiveness, Prognostication, and Reliability (RPR). Responsiveness TranslAGE evaluates biomarkers across 51 interventions, including pharmacological agents, supplements, lifestyle modifications, and medical procedures. This breadth enables identification of biomarkers that consistently capture changes in biological age across distinct mechanistic pathways, guiding trials from metabolic health to cellular senescence. Prognostication The platform integrates data from 50,000+ individuals across diverse populations to assess biomarkers’ ability to predict 40+ outcomes—frailty, cognitive decline, cardiovascular disease, and mortality. Unlike prior Western-centric studies, TranslAGE employs standardized metrics to enable cross-population and cross-outcome comparisons, ensuring global relevance. Reliability Utility depends on reproducibility. TranslAGE assesses: Technical reliability using DNA methylation datasets across Illumina 450K, Epic v1, and Epic v2 arrays. Biological reliability by quantifying variability from real-world factors such as circadian timing, stress, and diet—critical for clinical deployment. By uniting these dimensions in a standardized framework, TranslAGE enables shortlisting of biomarkers that are biologically informative, clinically practical, and globally generalizable. This positions the platform as a foundation for advancing the next generation of geroscience clinical trials.

